# Identification and characterization of the merozoite surface protein 1 (*msp1*) gene in a host-generalist avian malaria parasite, *Plasmodium relictum* (lineages SGS1 and GRW4) with the use of blood transcriptome

**DOI:** 10.1186/1475-2875-12-381

**Published:** 2013-10-30

**Authors:** Olof Hellgren, Megan Kutzer, Staffan Bensch, Gediminas Valkiūnas, Vaidas Palinauskas

**Affiliations:** 1 MEEL Department of Biology, Lund University, Lund, Sweden; 2 Institute for Evolution and Biodiversity, Westfälische Wilhelms-Universität Münster, Münster, Germany; 3 Institute of Ecology, Nature Research Centre, Vilnius, Lithuania

**Keywords:** Avian malaria, Merozoite surface protein 1, Genetic variation, *Plasmodium relictum*, SGS1, GRW4

## Abstract

**Background:**

The merozoite surface protein 1 (*msp1*) is one of the most studied vaccine candidate genes in mammalian *Plasmodium* spp. to have been used for investigations of epidemiology, population structures, and immunity to infections. However methodological difficulties have impeded the use of nuclear markers such as *msp1* in *Plasmodium* parasites causing avian malaria. Data from an infection transcriptome of the host generalist avian malaria parasite *Plasmodium relictum* was used to identify and characterize the *msp1* gene from two different isolates (mtDNA lineages SGS1 and GRW4). The aim was to investigate whether the *msp1* gene in avian malaria species shares the properties of the *msp1* gene in *Plasmodium falciparum* in terms of block variability, conserved anchor points and repeat motifs, and further to investigate the degree to which the gene might be informative in avian malaria parasites for population and epidemiological studies.

**Methods:**

Reads from 454 sequencing of birds infected with avian malaria was used to develop Sanger sequencing protocols for the *msp1* gene of *P. relictum*. Genetic variability between variable and conserved blocks of the gene was compared within and between avian malaria parasite species, including *P. falciparum*. Genetic variability of the *msp1* gene in *P. relictum* was compared with six other nuclear genes and the mtDNA gene cytochrome *b*.

**Results:**

The *msp1* gene of *P. relictum* shares the same general pattern of variable and conserved blocks as found in *P. falciparum*, although the variable blocks exhibited less variability than *P. falciparum*. The variation across the gene blocks in *P. falciparum* spanned from being as conserved as within species variation in *P. relictum* to being as variable as between the two avian malaria species (*P. relictum* and *Plasmodium gallinaceum*) in the variable blocks. In *P. relictum* the highly conserved p19 region of the peptide was identified, which included two epidermal growth factor-like domains and a fully conserved GPI anchor point.

**Conclusion:**

This study provides protocols for evaluation of the *msp1* gene in the avian malaria generalist parasite *P. relictum*. The *msp1* gene in avian *Plasmodium* shares the genetic properties seen in *P. falciparum*, indicating evolutionary conserved functions for the gene. The data on the variable blocks of the gene show that the *msp1* gene in *P. relictum* might serve as a good candidate gene for future population and epidemiological studies of the parasite.

## Background

There are more than 50 morphologically described species of *Plasmodium* that infect birds (i.e., causing avian malaria) throughout the world, but there may be several thousand evolutionary independent lineages that act as separate species on their avian hosts [1]. For over a decade molecular studies have been conducted on avian malaria. These were primarily based on cytochrome *b* diversity (different cytochrome *b* haplotypes are hereafter referred to as cyt *b* lineages) and in a few cases combined with a handful of other nuclear and mtDNA genes [1-3]. By combining published data on avian malaria parasites in communal databases, a vast lineage diversity, as well as remarkable host ranges and geographic spread of some parasite species has been observed [4]. One of the well-known, morphologically defined parasites, *Plasmodium relictum*, consists of several different lineages (e g, SGS1, GRW4, GRW11, LZFUS01), is found on all continents except Antarctica and is transmitted in both tropical and temperate regions [5]. One of the lineages (GRW4) belonging to this taxonomically defined species has caused major population declines when invading endemic bird fauna [6]. *Plasmodium relictum* also exhibits extraordinary levels of host generalism; the lineages SGS1 and GRW4 have been found in 50 bird species belonging to 15 different families and 53 species belonging to 17 families, respectively (MalAvi data base 2013-06-04 [4]). But how has this parasite spread around the globe and into so many bird species? In order to comprehend these processes there is a need for variable genetic markers as well as an understanding of the genetic variation of genes involved in the invasion stage of the host cells.

The vaccine candidate gene, merozoite surface protein 1 (*msp1*) is one of the more variable genes found in the human malaria parasite *Plasmodium falciparum*[7]. Within *P. falciparum* the msp1 gene encodes a 190 kDa protein that is cleaved into four polypeptides of different length (p83, p42, p38 and p30) during erythrocytic merogony (i.e., schizogony) [8]. These four peptides are bound together on the surface of the merozoite during adhesion to the host’s erythrocyte and are anchored to the parasite’s membrane via glycosylphosphatidylinositol (GPI) located in the p42 fragment [9]. During erythrocyte invasion, the p42 peptide is cleaved into two new polypeptides, p33 and p19, resulting in the loss of the merozoite’s peptide coat. Once inside the erythrocyte, p19 is all that remains of *msp1*[10]. Several studies have found that antibodies to the p19 peptide frequently occur in populations with high malaria prevalence and can be associated with immunity to the parasite [11,12]. Further, due to the high variability of the gene, *msp1* has frequently been used to infer population structures and understand the epidemiology of primate malaria parasites [7,13-15]. Apart from the different polypeptide fragments, the *msp1* gene has been divided into 17 different blocks based on variability of the amino acid composition. Of these 17 blocks, six are conserved, four are semi-conserved, six are variable and one block consists of repetitive elements [16].

Obtaining data on nuclear genes involved in the host invasion process has been notoriously difficult in avian malaria parasites due to the fact that the hosts (i. e., birds) have nucleated erythrocytes and the genome sizes between the host and parasite differ 52-fold. However, during erythrocytic merogony, (i.e., when the parasites undergo asexual reproduction in the blood stream and invade numerous new red blood cells (RBCs)), genetic activity in the parasites is high [17,18], thus making it possible to utilize mRNA sequencing in order to acquire sequence data of nuclear genes of the parasites [19].

By using expressed sequence data from a partially sequenced transcriptome of birds infected with the SGS1 lineage of *P. relictum*[19] it was possible to: 1) identify and develop Sanger sequencing protocols for *msp1* in *P. relictum* and, 2) investigate the degree to which the *msp1* gene in avian malaria parasites shares the characteristics of the *msp1* gene found in *P. falciparum*, which makes it a good candidate gene for future population studies and investigations focusing on the RBC invasion mechanisms across diverse malaria parasite species.

## Methods

### *msp1* identification and sequencing

**Figure 1:**
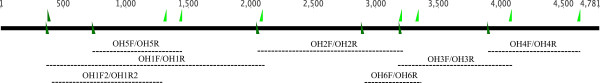
**Primer sites (Forward: dark green arrow, Reverse: light green arrows) along the *msp1 *gene.** Amplified fragments with corresponding primer pairs (Additional file 1) are represented by dashed lines.

*Plasmodium gallinaceum msp1* (AJ809338.1) was used as a reference sequence against which all raw reads from the partial transcriptome of SGS1 reads (SRA number SRR834578, [19]) were blasted against using Geneious 6.1. Reads with an E-value scored below 1e-10 were kept and used for potential primer sites. Primers were constructed using the primer design tool implemented in Geneious 6.1. Primers were tested and optimized using a sample of SGS1 (host is the Crossbill *Loxia curvirostra*) with high parasitaemia as well as a sample from a Great Reed Warbler (*Acrocephalus arundinaceus*) infected with GRW4. The lineage GRW4 also belongs to the morphologically defined species of *P. relictum*, but differs by 2.3% on the cyt *b* gene. Both samples originated from infection experiments in which the individuals were controlled for not having multiple infections using both microscopy and PCR methods [20]. For both of the samples, genomic DNA was extracted from a small blood sample using an ammonium acetate protocol according to Richardson *et al*. [21]. Primers were located according to Figure 1 and listed in Additional file 1. PCR reactions were performed in 10 μl reactions using a Qiagen multiplex PCR kit. For each sample 5 μl PCR ready reaction mix was used together with 2.6 μl ddH_2_O, 0.2 μl of each primer (10 μM) and 2 μl of DNA template of a concentration of 25 ng/μl. The PCR temperature profile was 95°C for 15 min followed by 35 cycles of 95°C for 30 sec, annealing temperature according to Additional file 1 for 90 sec and 72°C for 90 sec, the cycle was followed by a step of 72°C for 10 min. Positive amplifications were identified using 2 μl of the samples on a 2% agarose gel. PCR products from positive amplifications were precipitated and both the forward and reverse strands were sequenced using BigDye on an ABI 3100 sequencer.

**Table 1:** **Amino acid similarity (%) between different *Plasmodium *** **species/lineages**

**Block**	**X**	**Pg/SGS1**	**Pf/SGS1**	**Pg/Pf**	**SGS1/GRW4**	**Pf amino acid positions**	**Pg amino acid positions**
All		49.8	32.9	33.7	89.9	whole	whole
1	c	44.4	34.1**	46.4	ND	1–55	1–56
2	r	-	-	-	-	56–111	-
3	c	62.9	41.3	42.7	84.7*	112–313	57–255
4	v	39.1	28.1	21.9	86.4	314–345	256–277
5	c	62.9	60	74.3	97.1	346–380	278–312
6	v	44.9	25.8	25.4	93.7	381–609	313–536
7	sc	55.4	48.6	50	93.2	610–682	537–610
8	V^1^	36.1	14.4	16.3	84.7	683–779	611–717
9	sc	62.6	49.5	52.3	96.3	780–886	718–824
10	v	32.6	17.4	19.7	88.3	887–1024	825–953
11	sc	54.3	48.6	42.9	91.4	1,025–1,059	954–988
12	c	57.5	51.2	50.6	97.5	1,060–1,138	989–1,067
13	sc	52.2	45.2	39.3	96.4	1,139–1,222	1,068–1,149
14	v	20.8	14.9	12.7	76.1	1,223–1,288	1,150–1,220
15	c	44.6	42.2	37.1	89	1,289–1,316	1,221–1,308
16	v	54.8	27.9	29.1	98*	1,317–1,602	1,309–1,528
17	c	66	51.5	50.5	ND	1,603–1,702	1,529–1,622

*Plasmodiun gallinaceum* (Pg), *Plasmodium relictum* lineage SGS1 (SGS1), *Plasmodium relictum* lineage GRW4 (GRW4), *Plasmodium falciparum* (Pf) *Plasmodium yoelii* (Py).

Row X shows whether a block had been described previously as conserved (c), including repetitive motifs (r), variable (v) or semi-conserved (sc). The positions of the blocks are described in the last two columns after aligning *P. falciparum* (X05624.2) and *P. gallinaceum* (AJ809338.1).

**only partial sequence of SGS1, *only partial sequence of GRW4, ND: No sequence of the block in GRW4, - block is lacking for SGS1, GRW4 and Pg. Blocks were assigned according to Tanabe *et al*. [16]. ^1^According to Rich and Ayala this block is also containing repetitive sections [22].

For the SGS1 lineage, the end and beginning of the *msp1* gene was obtained using the 454 transcriptome reads [19]. (Reads can be found at NCBI Sequence Read Archive under project number: PRJNA196990; SRR834578).

Translated *msp1* sequences of SGS1 and GRW4 were aligned against *P. gallinaceum* (AJ809338.1) and *P. falciparum* (AL844508) using CLUSTAL W as implemented in Geneious 6.1.

### Genetic analysis

In *P. falciparum* the *msp1* gene has been divided into 17 different blocks depending on amino acid variability [16]. The ortholog blocks between *P. gallinaceum vs P. relictum* (SGS1), *P. gallinaceum vs P. falciparum* MAD20 isolate (CAA29112.2), *P. relictum* (SGS1) *vs P. falciparum* MAD20 isolate (CAA29112.2), *P. relictum* (SGS1) *vs P. relictum* (GRW4) and *P. falciparum* MAD20 isolate (CAA29112.2) *vs* K1 isolate (P04932) were evaluated in order to elucidate whether the *msp1* gene in avian *Plasmodium* lineages exhibit similar properties in variability as seen in *P. falciparum*. The properties of the different blocks were evaluated by percent amino acid similarity. A second goal was to identify which block of the *msp1* gene exhibited large variation in order to find sections of the gene that have the potential to be used for future population studies of avian *Plasmodium* parasites.

### Nuclear reference genes

In mammalian *Plasmodium* species, high variability in *msp1* in relation to other genes has been well documented [13,14,16]. However, the *msp1* gene in avian malaria parasites might have evolved under fundamentally different selection regimes than in mammalian malaria species due to differences in the physiology of the hosts. Moreover, *P. relictum* is an extreme host generalist [4], which might further contribute to a different selection regime compared to the host specialist *P. falciparum*. Therefore, in order to evaluate how variable the *msp1* gene is in relation to other nuclear genes in the *P. relictum* genome, six more PCR protocols for amplification of nuclear genes were developed. Transcriptome data of SGS1 (PRJNA196990; SRR834578) was used to identify primer sites for the genes; eukaryotic initiation factor-4A (eIF4A), Myosin A (MyoA), asparagine-rich antigen (Asp), skeletal protein IMC1-related (IMC1), transmission-blocking target antigen s230 precursor (Ps230) and a conserved *Plasmodium* spp. protein with unknown function (PF08_0073). For all genes except IMC1 and PF08_0073, nested PCR protocols were applied using the primers and annealing temperatures presented in Additional file 2. PCR reactions and PCR cycling profiles followed the protocol by Hellgren *et al*. [2].

Genetic variability as percent nucleotide differences was calculated between SGS1/GRW4 and SGS1/*P. gallinaceum*, for the sequenced nuclear genes, mtDNA cyt b (accession numbers *P. gallinaceum*: AB599930.1, SGS1: JX196867 and GRW4: AF254975) the full *msp1* gene and block 14 of the *msp1* gene as implemented in Geneious 6.1. Further, the sequence divergence of the cyt b and *msp1* block 14 between SGS1/GRW4, SGS1/*P. gallinaceum* was calculated using a Kimura 2-parameter model with uniform rates as implemented in MEGA 5.0.

### Phylogenetic analysis

A phylogenetic tree from the *msp1* gene was constructed using the translated sequences (due to the high variability at the nucleotide level at several of the different blocks) of 13 *Plasmodium* spp. (*P. gallinaceum* AJ809338.1, *Plasmodium chabaudi* AB379641.1, *Plasmodium malariae* FJ824669.1, *Plasmodium ovale wallikeri* KC137340.1, *Plasmodium ovale curtisi* KC137349.1, *Plasmodium fragile* AB444068.1, *Plasmodium vivax* AF435593.1, *Plasmodium knowlesi* AB444050.1, *P. falciparum* MAD20 isolate (X05624.2), *P. falciparum* K1 isolate (X03371.1), *Plasmodium reichenowi* (AJ786604.1), *P. relictum* (SGS1) KC969175 and *P. relictum* (GRW4) KC969176). The *msp1* gene consists of several repeat regions as well as highly variable regions, which makes alignments of the gene problematic. In order to reduce bias due to faulty alignments only conserved regions of the gene were used for the final phylogenetic analysis. For these regions (indicated with green bars) and alignment, see Additional file 3. The protein sequences were aligned using MUSCLE with eight iterations as implemented in Geneious ver. 6.1 The protein phylogeny was constructed using a Maximum Likelihood approach as implemented in PHYML [23] with a Blosum62 substitution model and a NNi Topology search. A consensus phylogeny with supporting bootstrap values was estimated using 1,000 iterations.

### Ethical approval

Care and handling of experimental animals was in accordance with the current laws of Lithuania (Lithuanian State Food and Veterinary Service, Ref. No. 2012/01/04-0221.

## Results

### Identification and characterisation of the *msp1* gene in *Plasmodium relictum*

With the use of a partially sequenced transcriptome of birds infected with *P. relictum* (SGS1) sufficient reads along the msp1 gene were identified in order to develop PCR protocols for Sanger sequencing of 4,273 bp of the total 4,869 bp length of the gene (*P. gallinaceum*). The protocols amplified *msp1* fragments for both the lineages SGS1 and GRW4. For SGS1, 319 bp at the start of the 3′end and 147 bp located in the last part of the 5′ end was identified and concatenated using raw sequence reads from the transcriptome [19]. It should be noted that the SGS1 lineage sequenced for the transcriptome originates from the same location as the Sanger sequenced SGS1 lineage in this study and are thus likely to exhibit identical sequences at the 3′ and 5′ ends. The resulting concatenated sequence length for SGS1 was 4,739 bp (Accession number: KC969175) and for GRW4 3,596 bp (Accession number: KC969176). The sequences were translated and the similarity at the amino acid level across the full SGS1 gene was 33.2% *vs P. falciparum* MAD20, 50.4% *vs. P. gallinaceum* and 89.9% *vs P. relictum* (GRW4).

### Similarity in block variability and identification of *msp1* (p19)

Within *P. falciparum*, the *msp1* gene has been defined based on 17 different blocks that show differences in amino acid variability. The corresponding blocks in *P. gallinaceum*, *P. relictum* (SGS1) and *P. relictum* (GRW4) were identified (for positions of blocks see Table 1). Both *P. gallinaceum* and *P. relictum* (SGS1) seem to lack block 2 completely, which is a region that exhibits large population variation between different strains of *P. falciparum*. For *P. relictum* (GRW4), it was not possible to sequence the 5′ end of gene and the absence of block 2 could not be verified.

**Figure 2:**
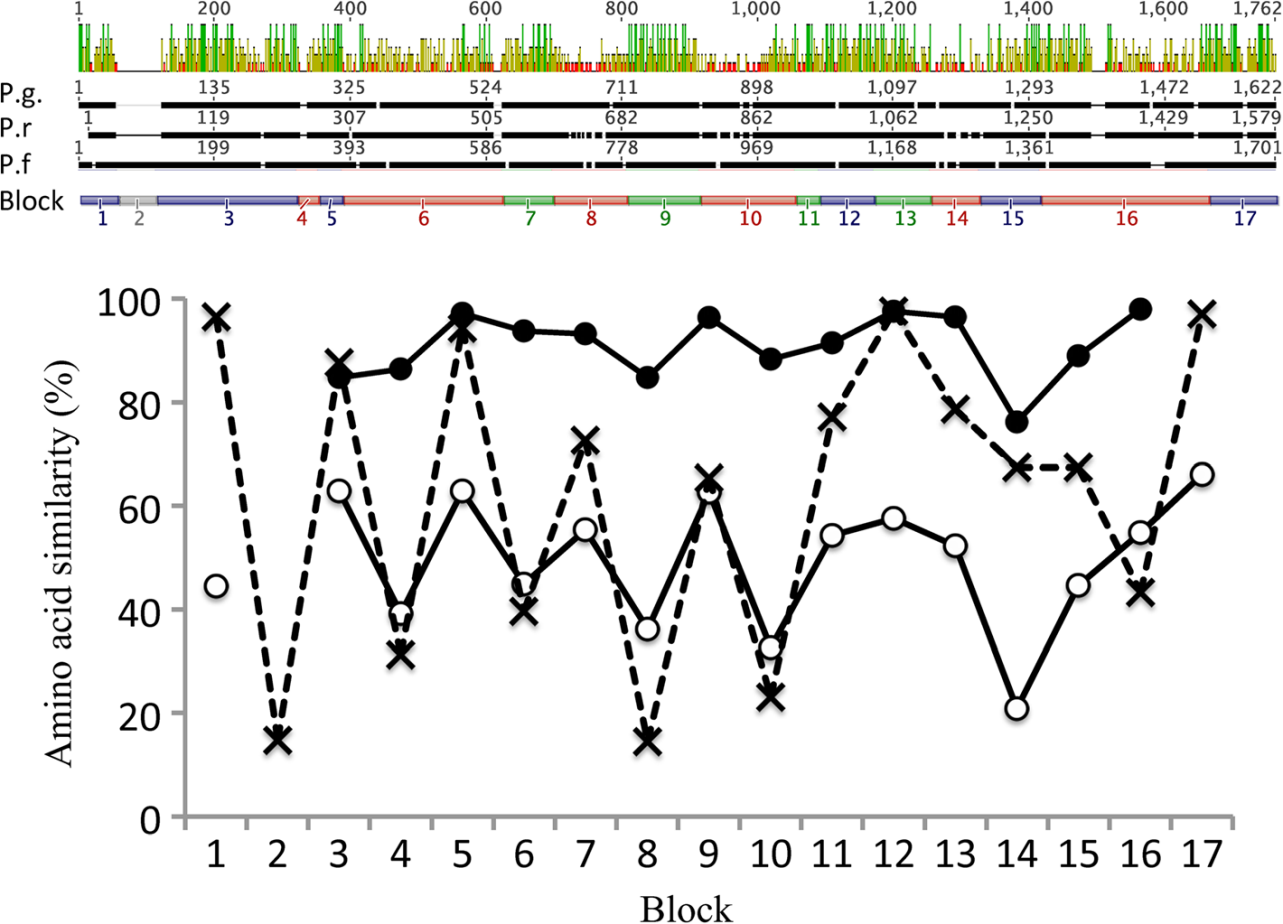
**Aligned amino acid sequences of *Plasmodium gallinaceum *(Pg), *Plasmodium relictum *lineage SGS1 (Pr) and *Plasmodium falciparum *(Pf).** Bars on top represent amino acid similarities: green: 100% identity, brown >30 < 100%, red <30% similarity. Blocks 1–17 are assigned according to Tanabe *et al*. [16] [compare Table 1 legend], blue: conserved, white: repetitive region; red, variable and green: semi-conserved region. Amino acid similarity (%) for the different block; solid line with filled circles: *P. relictum* (SGS1 *vs* GRW4), solid line with open circles: *P. gallinaceum* (Pg) *vs P. relictum* (SGS1) and dashed line with X: *P. falciparum* MAD20 isolate (X05624.2) *vs* K1 isolate (X03371.1).

**Figure 3:**
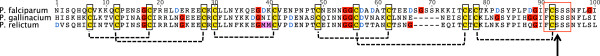
**Amino acid alignment of the *msp1*-p19 fragment between *P. relictum *(SGS1) and *P. falciparum *(MAD20 isolate X05624.2).** Dashed lines with black boxes represent conserved cysteine motifs with corresponding disulphide bonds forming, for the region, the two characteristic epithelia growth factor-like epitopes. Arrow indicates the anchor point in the merozoite membrane with the conserved “FCSS” motif surrounding the anchor point (red box).

Comparing the variability of the different blocks between *P. falciparum* (MAD20 *vs* K1), *P. gallinaceum vs P. relictum* (SGS1) and *P. relictum* (SGS1 *vs* GRW4) showed similar patterns of which blocks being the most variable (Table 1, Figure 2). For the more distantly related avian malaria species (*P. gallinaceum vs P. relictum* (SGS1)) the pattern across the different blocks was similar to the *P. falciparum* MAD20 *vs* K1 comparison with the same blocks being variable or conserved except for block 16, which did not seem to be as variable as in *P. falciparum* (Table 1, Figure 2). For the close lineage comparison (*P. relictum* (SGS1) *vs P. relictum* (GRW4)), again similar patterns of variability occur, although clearly showing less variation. The blocks that show the greatest variability are blocks 14, 8, 10 and 3, with block 14 being the most variable, which is comparable to variation found in *P. falciparum*. The most conserved regions between the avian malaria lineages were 5, 7, 9, 12, 13, 16 and 17 (Table 1, Figure 2). One of the most conserved regions between SGS1/*P*. *gallinaceum* and SGS1/*P. falciparum* was block 17, which includes the region encoding the p19 peptide (Figure 3) that anchors to the merozoite cell membrane and is maintained during RBC invasion.

### *msp1* variability in relation to other nuclear genes

**Figure 4:**
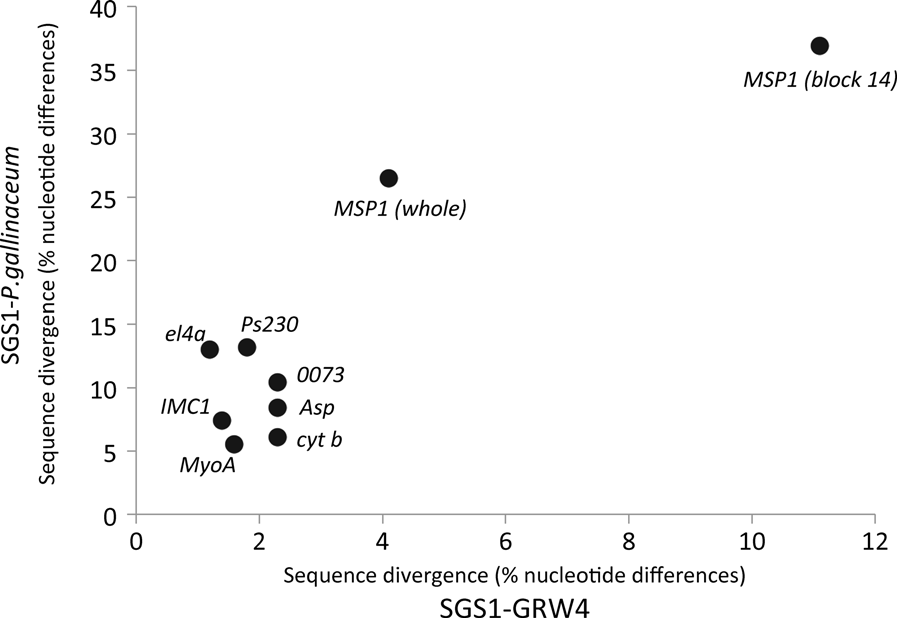
**Comparison of nuclear sequence divergence (%) for six nuclear genes, the cytochrome b gene, the whole msp1 gene and block 14 of the msp1 gene between *P. relictum *** **(SGS1) and *P. relictum *** **(GRW4) (X-axis) and between *P. relictum *** **(SGS1) and *P. gallinaceum *** **on Y-axis.**

The genetic dissimilarity between *P. relictum* (SGS1) and *P. gallinaceum* based on six nuclear genes, not including *msp1*, (Accession numbers KF026619-KF026630) and one mitochondrial gene (cyt b) ranged between 5.5 and 13.2% and 1.2 and 2.3% between lineages SGS1 and GRW4. For the whole *msp1* gene, the corresponding values were 26.5 and 4.1%. For *msp1* block 14, the same comparisons were as high as 36.9 and 11.1%, respectively (Figure 4). Using a Kimura 2-parameter model, the calculated rate of molecular change between *P. relictum* (SGS1 and GRW4) was 0.023 for cyt *b* and 0.122 for *msp1* block 14. The same comparison between *P. relictum* (SGS1) and *P. gallinaceum* gave rates of 0.063 and 0.648 respectively. When taken together these rates indicate a 5–10 fold higher rate of molecular change in the *msp1* block 14 region compared to the rate observed in the cyt *b* gene.

### Phylogenetic analysis of ten *Plasmodium* spp

**Figure 5:**
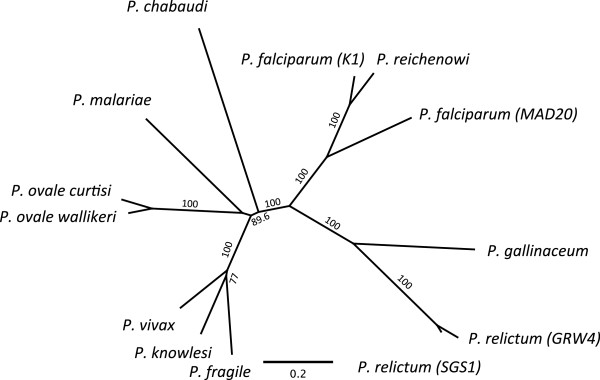
**Maximum likelihood consensus phylogeny of the translated msp1 gene from 12 different malaria species.** Numbers on the branches represent bootstrap values based on 1,000 iterations.

The ML tree of the protein alignments showed that avian *msp1* forms a well-supported, monophyletic group that has diverged significantly from mammalian *Plasmodium* species (Figure 5), with the two *P. relictum* lineages being less divergent than the *P. falciparum* isolates (isolates MAD20 and K1).

## Discussion

Malaria parasites (i.e., *Plasmodium* spp.) are found to infect a huge diversity of hosts, including birds, mammals and reptiles. By understanding how genes taking part in the invasion of host cells and tissues have evolved across this vast host range might help us to identify genes and processes that are evolutionarily constrained or are of importance for limiting a parasite to one or several host species. One such gene that is involved in the invasion process is *msp1*. The *msp1* gene encodes for one of the most abundant proteins on the surface of the merozoite (the stage of the parasite that invades the RBC of the host), and is thought to be involved in the initial adhesion to the RBC. This gene is, therefore, highly important for the understanding of invasion biology of malaria parasites as well as for the understanding of how genes involved in the invasion of the host have evolved across different host ranges.

This study identified and investigated the *msp1* gene originating from two different cyt *b* lineages of the avian malaria parasite *P. relictum* (SGS1 and GRW4). The phylogeny of the *msp1* peptides placed the avian malaria parasites into a strongly supported monophyletic cluster. Two *P. relictum* lineages showed a genetic divergence below that found between different *P. falciparum* isolates MAD20 and K1. The genetic distance between *P. relictum*/*P. gallinaceum* exceeded the genetic distance seen between *P. falciparum* (K1)/*P. reichenowi* (Figure 5).

Similar patterns of conserved and variable blocks were found across the gene when comparing the block variability observed between *P. relictum (SGS1)*/*P. gallinaceum* with the variability between the two different *P. falciparum* isolates (Figure 2). In addition, when comparing the *msp1* haplotypes of *P. relictum* (SGS1 and GRW4), the same blocks that have been defined as being variable in *P. falciparum*[16] were also the most variable in this comparison. These similarities might indicate that the properties and function of the avian malaria *msp1* gene is similar to the mammalian malaria *msp1*. However, when comparing the degree of variation in the different blocks between the two different *msp1* isolates of *P. falciparum* (MAD20/K1) some of the conserved blocks (block 3, 5 and 12, Figure 2) were as conserved as between the two *P. relictum* lineages (representing the same morphological defined species), while some of the variable blocks (block 4, 6, 8, 10, and 16, Figure 2) showed variability that exceeded that found between two distinctly different avian malaria parasite species (*P. relictum*/*P. gallinaceum*). This variability exceeded that of *P. relictum*/*P. gallinaceum* although the overall genetic distance between the *P. relictum*/*P. gallinaceum msp1* gene is bigger than that between *P. falciparum (K1)*/*P. reichenowi* (Figure 5). The differences in the degree of within species variability at the variable blocks might indicate different selection pressures acting upon the different regions in *P. relictum* compared to *P. falciparum*. However, in order to make comparisons between the two species and to know how and in what direction selection has acted upon the avian malaria gene, there is a need for larger population studies of the *msp1* gene. Larger population samples are needed in order to pinpoint sections of stabilising versus diversifying selection as well as being able to compare the degree of allelic polymorphism between the two.

Molecular characterization of avian malaria strains has, to date, mainly utilized cyt *b* sequences [2,4,24], together with a handful of nuclear genes, of which none show nuclear variation below the defined cyt *b* lineages [1,3]. In order to understand the epidemiology of avian malaria parasites that have large geographical transmission areas [4,25] as well as broad host ranges [26], there is a need for more variable nuclear markers. Although *msp1* block 2, which is often used for population studies in human malaria species, seems to be absent in avian malaria, other parts of the gene might well serve as useful molecular markers for inferring population structures below the level of cyt *b* lineages, in avian malaria. One such marker might be *msp1* gene block 14. Block 14 has diversified 5.3 and 10.2 times faster between *P. relictum* lineages SGS1/GRW4 and SGS1/*P. gallinaceum*, respectively, compared to the divergence rate of the cyt *b* gene. It is important to note that the block diversification rate observed in Block 14 in the current study may change when including more isolates and lineages of *P. relictum*. Still, it might serve as a good starting point for future studies wishing to examine the epidemiology and population structure of *P. relictum*.

During merozoite formation the *msp1* peptide is anchored to the parasite membrane via glycosylphosphatidylinositol (GPI) located at C-terminus of the protein. The GPI anchors are the last remaining part of the peptide (p19) during erythrocyte invasion. For *P. relictum* (SGS1), it was possible to identify the full 3′ end of the peptide; within this region the GPI anchor point was fully conserved at the amino acid level between *P. falciparum*, *P. gallinaceum* and *P. relictum* (SGS1) with the characteristic “FCSSS” motif (Figure 3). The “FCSSS” motif has been found to be extremely conserved across almost all investigated species of malariae parasites [27] and this was also the case when including more lineages of avian malariae species. In the p19 section of the avian malaria parasite peptides, it was also possible to identify the two epidermal growth factor-like domains (EGF), based on the six conserved characteristic cysteine residues [28] (Figure 3). In *P. falciparum*, the cysteine residues form disulphide bonds, which create a conserved structure similar to that of the epidermal growth factor-like domains. These conserved epitopes are acted upon by the host’s immune system through antibodies that provide the host with protective immunity against malaria. To some extent, antibodies against the p19 epitopes can give the host cross-immunity to other malaria strains. In the bird-malaria parasite system it is common that bird species can be infected with a broad diversity of different malaria species and lineages [29-31]. Within this system, *msp1*-p19 would be a promising target for investigating cross-immunity between different species and changes of this cross-immunity depending on the phylogenetic distance of the parasite. Further, *P. relictum* has caused large population declines and mortality in endemic host communities, when introduced into formerly parasite-free areas (e.g., Hawaii) [32-34]. Here, *msp1*-p19 could be used as a candidate gene to investigate whether individual birds that survive malaria infection carry antibodies against this peptide, in order to examine whether diverse host species target the same structures and pathways in the parasite when fighting the disease.

## Competing interests

The authors declare that they have no competing interests.

## Authors’ contributions

OH carried out the molecular work on the *msp1* gene, designed the study, drafted the manuscript. MK developed and carried out the molecular work on the nuclear genes excluding *msp1*. VP and GV isolated *Plasmodium* parasite strains, designed and conducted bird infection experiments from which samples for molecular work were used. OH, MK, GC, VP, and SB all participated in interpreting the data and made significant contribution to drafting the manuscript. All authors read and approved the final manuscript.

## Supplementary Material

Additional file 1 **Primer sequences and annealing temperatures for sequencing of the *msp1* gene of *Plasmodium relictum*.** Click here for file

Additional file 2 **Primer sequences and annealing temperatures for sequencing of seven nuclear genes of *Plasmodium relictum*.** Click here for file

Additional file 3 **Alignment of the *Plasmodium *species/lineages used for the phylogeny of Figure **5**.** Sections underlined with green bars are the sections used in the analysis. Click here for file
