# Supplementary material for: Identification and characterization of the merozoite surface protein 1 (msp1) gene in a host-generalist avian malaria parasite, Plasmodium relictum (lineages SGS1 and GRW4) with the use of blood transcriptome
Source: Malar J. 2013 Oct 30;12:381. doi: 10.1186/1475-2875-12-381 (PMC3827925; doi:10.1186/1475-2875-12-381)
Supplement: Additional file 1 — Primer sequences and annealing temperatures for sequencing of the msp1 gene of Plasmodium relictum . [file 1475-2875-12-381-S1.docx]

**Additional file 1 Primer sequences and annealing temperatures for sequencing of the *msp1* gene of *Plasmodium relictum***

| **Primer** |  | **Seq (as ordered)** | **Annealing**  **temp. (°C)** |
| --- | --- | --- | --- |
| msp1g_OH1F | Forward | 5'-AAGATATGTGCATGCGCGATGGTT-3' | 54 |
| msp1g_OH1R | Reversed | 5'-TCTTGCTTCTTCTACAGGATTTTCT-3' |  |
|  |  |  |  |
| msp1g_OH2F | Forward | 5'-ACCAGAACAACAAAGTTCAGGAAA-3' | 54 |
| msp1g_OH2R | Reversed | 5'-TTCTTCCGTTTCACTTAATGCTCT-3' |  |
|  |  |  |  |
| msp1g_OH3F | Forward | 5'-TAGAGCATTAAGTGAAACGGAAGA-3' | 54 |
| msp1g_OH3R | Reversed | 5'-AGGAAGAAGTTTTTCATCCTGTGA-3' |  |
|  |  |  |  |
| msp1g_OH4F | Forward | 5'-AAGTGATAGAACCAGAAACAAGAA-3' | 52 |
| msp1g_OH4R | Reversed | 5'-TTTGCATTCACATCACATCCACCA-3' |  |
|  |  |  |  |
| msp1_OH5F | Forward | 5'-ATGAAGATATAAAACCATTGCTTGA-3' | 52 |
| msp1_OH5R | Reversed | 5'-ACTTACGTTGCCCCTCTAATTTTT-3' |  |
|  |  |  |  |
| msp1_OH6F | Forward | 5'-AATGAAACTTTACGTTATCGAGCT-3' | 52 |
| msp1_OH6R | Reversed | 5'-CCATTTGCATCATTAGATTCACCA-3' |  |
|  |  |  |  |
| msp1g_OH1F2 | Forward | 5'-GCGCGATGGTTGCAAAATACCATT-3' | 55 |
| msp1g_OH1R2 | Reversed | 5'-GTTCTTGAAGAGCATTAGCGTGTT-3' |  |
