# Supplementary material for: Identification and characterization of the merozoite surface protein 1 (msp1) gene in a host-generalist avian malaria parasite, Plasmodium relictum (lineages SGS1 and GRW4) with the use of blood transcriptome
Source: Malar J. 2013 Oct 30;12:381. doi: 10.1186/1475-2875-12-381 (PMC3827925; doi:10.1186/1475-2875-12-381)
Supplement: Additional file 2 — Primer sequences and annealing temperatures for sequencing of seven nuclear genes of Plasmodium relictum . [file 1475-2875-12-381-S2.docx]

| **Gene** | **Forward (F) and Reverse (R) Primers** | **Annealing Temp (°C)** |
| --- | --- | --- |
| ApiAP2 | ACCAGGGGTACGATTTAACCCCAA (F outer) | 56 |
|  | TGTTGCACCAGCTTGCTCTGCT (R outer) |  |
|  | GCAGGTGCAAGATGTAAGCCAATGT (F inner) | 56 |
|  | CCCGATTGACCAATAAGCAAGCCA (R inner) |  |
|  |  |  |
| eIF4a | TTGCCAAGCATTAATCCTAGCTCCAACTC (F outer) | 57 |
|  | AGTTCCTCCAACACAAGCGTGACA (R outer) |  |
|  | GCATTAATCCTAGCTCCAACTCGTG (F inner) | 50 |
|  | CCAACACAAGCGTGACATTTTAC (R inner) |  |
|  |  |  |
| MyoA | CATTCACACCATCTACATCTGTT (F outer) | 50 |
|  | TCGATCCACCAGGAATAGGAAC (R outer) |  |
|  | ATGGTATGACCTTTCATTATCATC (F inner) | 46 |
|  | ACCACAGCTCTTCCTCTTATTGTTGC (R inner) |  |
|  |  |  |
| Ps230 | AGTTGAACTATTCGACTTGGCCTGT (F outer) | 52 |
|  | TGAGCATACCTGTGAAATTGAAGCA (R outer) |  |
|  | TCCACTTGGGCATGCAAATCCT (F inner) | 52 |
|  | ATGCGACAACAGTGCTACACAA (R inner) |  |
|  |  |  |
| IMC1 | GTACCAGAAGTGAACTGCCCAGA (F) | 64 |
|  | CCTGTGTTCCGCTTCTCATGGT (R) |  |
|  |  |  |
| PF08_0073 | ACATTGCAGTCATCTTTACCCCAAGC (F) | 63 |
|  | AGACCCCACCTGCAAGTGCAAA (R) |  |

**Additional file 2.** **Primer sequences and annealing temperatures for sequencing of seven nuclear genes of *Plasmodium relictum***
