# Supplementary material for: Identification and characterization of the merozoite surface protein 1 (msp1) gene in a host-generalist avian malaria parasite, Plasmodium relictum (lineages SGS1 and GRW4) with the use of blood transcriptome
Source: Malar J. 2013 Oct 30;12:381. doi: 10.1186/1475-2875-12-381 (PMC3827925; doi:10.1186/1475-2875-12-381)

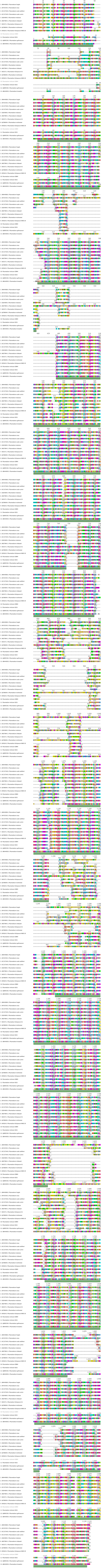

Supplement: Additional file 3 — Alignment of the Plasmodium species/lineages used for the phylogeny of Figure 5. Sections underlined with green bars are the sections used in the analysis. [file 1475-2875-12-381-S3.pdf]
